# Characterization of alfalfa virus F, a new member of the genus *Marafivirus*

**DOI:** 10.1371/journal.pone.0203477

**Published:** 2018-09-04

**Authors:** Lev G. Nemchinov, Sarah François, Phillipe Roumagnac, Mylène Ogliastro, Rosemarie W. Hammond, Dimitre S. Mollov, Denis Filloux

**Affiliations:** 1 USDA-ARS, Molecular Plant Pathology Laboratory, Beltsville MD, United States of America; 2 INRA, UMR DGIMI, Montpellier, France; 3 CIRAD, UMR BGPI, Montpellier, France; 4 BGPI, CIRAD, INRA, Montpellier SupAgro, Univ Montpellier, Montpellier; 5 USDA-ARS, National Germplasm Recourses Laboratory, Beltsville MD, United States of America; Oklahoma State University, UNITED STATES

## Abstract

Viral infections of alfalfa are widespread in major cultivation areas and their impact on alfalfa production may be underestimated. A new viral species, provisionally named alfalfa virus F (AVF), was identified using a virion-associated nucleic acid (VANA) metagenomics-based approach in alfalfa (*Medicago sativa* L.) samples collected in Southern France. The nucleotide sequence of the viral genome was determined by *de-novo* assembly of VANA reads and by 5’/3’ RACE with viral RNA extracted from enriched viral particles or with total RNA, respectively. The virus shares the greatest degree of overall sequence identity (~78%) with *Medicago sativa* marafivirus 1 (MsMV1) recently deduced from alfalfa transcriptomic data. The tentative nucleotide sequence of the AVF coat protein shares ~83% identity with the corresponding region of MsMV1. A sequence search of the predicted single large ORF encoding a polyprotein of 235kDa in the Pfam database resulted in identification of five domains, characteristic of the genus *Marafivirus*, family *Tymoviridae*. The AVF genome also contains a conserved “marafibox”, a 16-nt consensus sequence present in all known marafiviruses. Phylogenetic analysis of the complete nucleotide sequences of AVF and other viruses of the family *Tymoviridae* grouped AVF in the same cluster with MsMV1. In addition to 5’ and 3’ terminal extensions, the identity of the virus was confirmed by RT-PCRs with primers derived from VANA-contigs, transmission electron microscopy with virus-infected tissues and transient expression of the viral coat protein gene using a heterologous virus-based vector. Based on the criteria demarcating species in the genus *Marafivirus* that include overall sequence identity less than 80% and coat protein identity less than 90%, we propose that AVF represents a distinct viral species in the genus *Marafiviru*s, family *Tymoviridae*.

## Introduction

Alfalfa (*Medicago sativa* L.) is the most extensively cultivated forage legume in the world and the fourth most widely grown crop in the U.S. Alfalfa productivity has often been limited by various biotic and abiotic components in the ecosystem [1,2]. Minimizing these losses is a major area of concern in the alfalfa industry. Traditionally, viral infections of alfalfa are considered diseases of limited importance even though they are widespread in major cultivation areas and their contribution to the severity of complex infections involving multi-pathogens is poorly known. More recently, emerging viral diseases of alfalfa have been described with the potential to cause serious yield losses. These include a rhabdovirus, diagnosed in alfalfa plants displaying multiple abnormalities [3]; a new enamovirus from Argentina, alfalfa enamovirus-1 (AEV-1), detected in alfalfa plants showing dwarfism symptoms [4]; an AEV isolate from Sudan, designated AEV-2 [5]; a new species of the family *Alphaflexiviridae* discovered in alfalfa samples exhibiting chlorosis and stunting [6]; and alfalfa leaf curl virus found in plants displaying leaf curling symptoms [7].

Marafiviruses are positive strand RNA viruses transmitted by leafhoppers in a persistent manner. Their genome has a large open reading frame (ORF) of about 6.3–6.8 kb in size, encoding a precursor polypeptide composed of the replication-associated proteins and one of the two forms of the coat protein (CP) found in the virions [8,9]. The type species of the genus *Marafivirus* is *Maize rayado fino virus* (MRFV) [10]. Until very recently, marafiviruses were not reported to infect alfalfa. In 2018, the nucleotide sequence of the proposed Medicago *sativa* marafivirus 1 (MsMV1) was deduced from alfalfa transcriptomic data by Kim et al., [11]. These alfalfa transcriptomic data were previously deposited to NCBI and published by a different group of authors [12]. The reported sequence of MsMV1 appears to be missing the 5’ terminus and there is no evidence that terminal extensions were performed to confirm its sequence.

In this work, we report identification and the complete genomic sequence of a new alfalfa marafivirus, obtained by *de-novo* assembly of VANA reads and by 5’/3’ RACE with total RNA extracted from infected plants. The identity of the virus reported in this study was also confirmed by RT-PCRs with primers derived from VANA-contigs, transmission electron microscopy observations, and transient expression of the viral coat protein gene using a heterologous plant virus-based vector. Based on the current species demarcation criteria, the virus, provisionally named alfalfa virus F (AVF), represents a distinct species in the genus *Marafiviru*s, family *Tymoviridae*.

## Materials and methods

### Plant samples

From 2010 to 2016, 33 alfalfa leaf samples were collected in Southern France to explore their virome using metagenomics approaches. The owners of the alfalfa fields where the plants were sampled gave permission to conduct the study on their sites. Samples were collected randomly, regardless of any potential symptoms. Two alfalfa plants, from which leaf samples were collected, were maintained *in vivo* in insect proof growth chambers at Cirad, International Campus of Montpellier, France.

### Virion-associated nucleic acids (VANA) metagenomics-based approach

Thirty-three alfalfa samples were processed using the VANA-based 454 pyrosequencing approach exactly as described in François et al. [13]. PCR amplicon libraries were sequenced on an Illumina MiSeq platform as 2x300 bp paired-end reads (Beckman Coulter Cogenics, Morrisville, NC, USA). Bioinformatics analyses were performed as described in François et al. [13]. Briefly, read cleanups and corrections were performed using the CutAdapt version 1.9 program (https://cutadapt.readthedocs.io/en/stable/) and *de-novo* assemblies were generated using SPAdes 3.6.2 software (http://cab.spbu.ru/software/spades/).

### Total RNA extraction, RT-PCRs, cloning, 5’/3’ RACE, and virion-associated nucleic acids (VANA) extraction

For 5’ and 3’ RACE systems and RT-PCR assays, total RNA was extracted from one alfalfa leaf sample collected in a commercial alfalfa field at Prades-le-Lez, Southern France (GPS location: 43°42'16.44"N 3°51'48.31"E) using TRIzol RNA isolation reagent as described by the manufacturer (ThermoFisher Scientific, Waltham, MA). RT-PCRs were performed with total RNA employing the SuperScript RT-PCR system per the manufacturer’s directions (ThermoFisher Scientific). RT-PCR products were either sequenced directly or cloned into the pCRII-TOPO vector with dual promoter (ThermoFisher Scientific) for sequencing.

The 5’terminus of the virus was determined using the SMARTer® RACE 5'/3' Kit (Takara Bio USA, Inc., Madison. WI) with the genome-specific primer LN529 (Table 1). The 3’ terminal sequence was obtained with the 3’ RACE system (ThermoFisher Scientific) and genome-specific primers LN522 and LN523 (Table 1).

**Table 1:** Primers used for 5’RACE, 3’RACE and RT-PCR.

Primer name	Primer position	Primer sequence, 5’ to 3’
LN522	5517–5536	CGCCAACTTCGACTTCTTCT
LN523	5776–5797	CTTGACTTCGCTCCTCTTGC
LN529	902–924	GGATTCAGGAGGGACAACTAAAG
LN568	5890–5919	gatatcATGGCCCTCTCTGCTATTGAA
LN569	6584–6603	gatatcCTATTTGGAAGGGGTGGCGG

### Phylogenetic analysis

A dataset consisting of the complete nucleotide sequences of alfalfa virus F and 12 ICTV-approved and tentative members of the genus *Marafivirus* was assembled. These sequences were aligned and phylogenetic tree built with the CLC Genomics Workbench software (Qiagen Inc) using neighbor-joining algorithm, Jukes-Cantor distances and 1000 bootstrap replicates.

### Cloning into a potato virus X (PVX)-based vector, transcript preparation and inoculation of plants

RT-PCR products amplified with primers LN568-LN569 were cloned into the pCR TOPO II vector (ThermoFisher Scientific), digested with *Eco*RV, gel-purified and sub-cloned into the *Eco*RV-linearized PVX-based vector pP2C2S [14], (pP2C2S obtained from D. Baulcombe, Sainsbury Laboratories, Norwich, England). pP2C2S plasmids were linearized with *Spe*I, and capped transcripts were generated from cDNA clones using Ambion’s T7 mMessage Machine kit (ThermoFisher Scientific). The transcripts were mechanically inoculated onto fully expanded leaves of *Nicotiana benthamiana*.

### Transmission electron microscopy

For transmission electron microscopy (TEM), viral particles from infected alfalfa tissues were partially purified using a protocol developed for *Poinsettia mosaic virus* [15]. For TEM observation of virus-like particles generated via the PVX vector in *N*. *benthamiana* plants, samples were processed as described in [6]. Virus captured on the TEM grids was stained with 1% phosphotungstate (PTA) solution. The grids were examined in a Hitachi H-7700 Electron Microscope at the Electron and Confocal Microscope Unit, Beltsville Agricultural Research Center.

## Results

### Metagenomics-based discovery of a novel marafivirus from alfalfa plants collected in Southern France

*De novo* assemblies of VANA reads revealed the presence of contigs sharing similarities with marafiviruses in four alfalfa samples, including one plant sample from Prades-le-Lez (Montpellier region) and three plant samples from the Rhône delta region. Interestingly, one of these three plants, originating from the Rhône delta region, has been maintained at the Cirad laboratory and is not exhibiting any visible symptoms that would differentiate it from healthy plants (S1 Fig).

### Nucleotide sequence and genome organization

To confirm the identity of the sequenced sample as *Medicago sativa* species, all transcripts generated by the VANA metagenomics approach [13] were analyzed by BLASTn. Most of the eukaryotic transcripts with the length > 300 nucleotides (nt) and identity >90% aligned to the species in the subfamily *Papilionoideae*, genus *Medicago* thus validating the host as *Medicago sativa*. Assembly of the raw data reads resulted in an incomplete viral genome lacking 5’ and 3’-end sequences of the mRNA, including the coat protein. After missing sequences were generated using the Takara Smarter 5’/3’ RACE protocol and the ThermoFisher Scientific 3’RACE system, respectively, the full-length monopartite viral genome consisting of 6,818 nts was assembled and functionally annotated.

**Fig 1:**
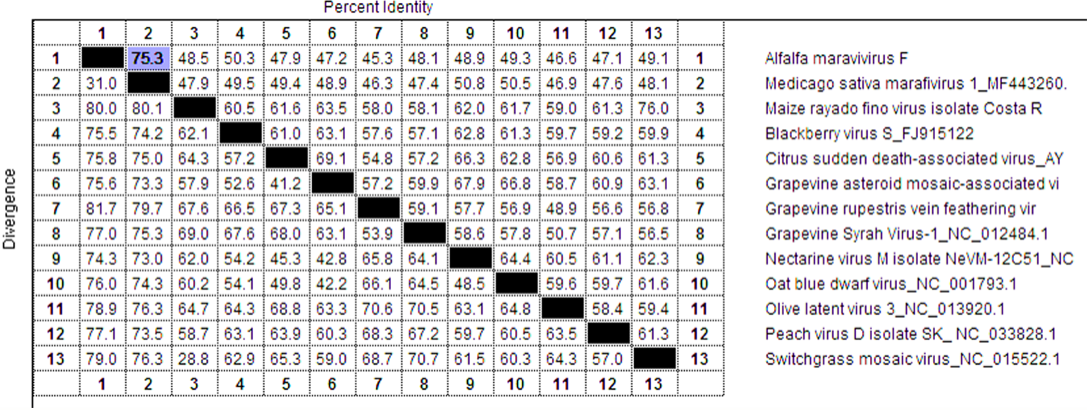
Multiple nucleotide sequence alignment between complete genomes of AVF and other marafiviruses. Alignment generated using DNASTAR software, MegAlign tool with ClustalW algorithm. Percent identity for each pair of sequences is shown on the upper right side and percent diversity (calculated by comparing sequence pairs in relation to the phylogeny reconstructed by MegAlign) is shown on the lower left side. Percent identity between AVF and MsMv1 (75.3%) highlighted in blue.

On the nucleotide level, AVF has 78% identity with the newly identified and proposed alfalfa marafivirus MsMV1 [11], (BLASTN query coverage 80%, E-value = 0.0, accession № MF443260.1). The next closest species from the genus *Marafivirus*, per PASC tool [16], is oat blue dwarf virus isolate OBDV2r (50.2% identities, accession № GU396990), a member of the genus *Marafivirus*. Multiple nucleotide sequence alignment using the MegaAlign tool and ClustalW algorithm of the DNAStar package (DNASTAR Inc., WI, USA) generated even lower identity between the complete genomes of AVF and MsMV1 (75.3%); identity with other viruses of the genus was at the 50% level or less (Fig 1). Therefore, based on the nucleotide sequence alignment of the compete AVF genome and criteria demarcating species in the genus *Marafivirus* that include overall sequence identity less than 80% [8], AVF represents a distinct viral species in the genus *Marafiviru*s, family *Tymoviridae*.

AVF encodes a single precursor polyprotein 2130 amino acids (aa) in length. In BLASTP query, the AVF polyprotein was 82% identical to the polyprotein of MsMV1 (99% coverage, E-value = 0.0, accession № ATJ00054.1). The next closest species was *Nectarine marafivirus M* (49% identities, 98% query cover, E-value = 0.0, accession № YP_009222597.1). The Pfam database search and analysis of the AVF polyprotein (http://pfam.xfam.org/) resulted in five significant Pfam-A matches corresponding to five viral domains characteristic for the genus *Marafivirus*, family *Tymoviridae*: viral methyltransferase (PF01660; E-value 7.9e-55), tymovirus endopeptidase (PF05381; E-value 3.8e-19), viral (superfamily 1) RNA helicase (PF01443; E-value 5.3e-52), RNA dependent RNA polymerase (PF00978; E-value 5.9e-13) and tymovirus coat protein (PF00983; E-value 4.2e-20).

**Fig 2:**
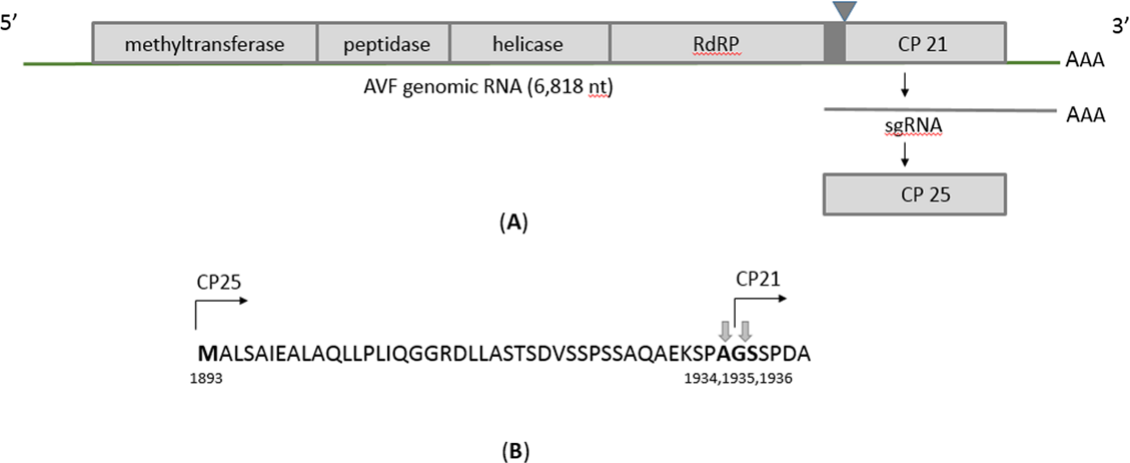
(**A**) Tentative translation strategy for AVF coat proteins. The open reading frames are drawn as rectangles. The black box indicates location of the “marafibox” subgenomic promoter; sgRNA, subgenomic RNA; CP21, major coat protein, molecular weight 21kDa; CP25, minor coat protein, molecular weight 25.2kDa; Triangle indicates putative cleavage site for viral or cellular proteases to produce CP21. (**B**) The amino acid sequence of the AVF CP region showing putative location of the methionine initiation codon for CP25 (1893 aa) and putative cleavage sites for CP21 between the amino acid residues Ala_1934_ and Gly_1935_ or Gly_1935_ and Ser_1936_ (arrows).

CP-encoding sequences in marafiviruses are in the same reading frame with the polyprotein ORF and are located at its 3’terminal end. Marafivirus particles were reported to contain so called major and minor CPs, of about 21kDa and 25kDa, respectively, that differ by an amino terminal extension in the minor CP and are found in the virus particles in molar ratios of approximately 3:1 (major CP21 vs minor CP25) [17,18]. However, it appears that unlike other marafiviruses, AVF as well as MsMV1, do not have a second initiation codon (Met) for the coding region of the major CP protein (CP21) and only encode methionine for the minor CP25 (5890–6603 nt; 1893–2130 aa). In this case, a possible strategy to produce two CPs could be a direct translation of the subgenomic RNA for the minor CP25 (rather than for CP21) and posttranslational cleavage of the larger precursor to produce the major protein CP21 (Fig 2A), [18]. A putative cleavage site to produce CP21 could be located downstream of the CP25 N-terminal end, speculatively between the amino acid residues Ala_1934_ and Gly_1935_ or Gly_1935_ and Ser_1936_ (Fig 2B).

The nucleotide sequence of the AVF CP25 displays 80% identity with the MsMV1 polyprotein gene (query cover 100%, E-value = 4e-142; accession № MF443260.1). On the amino acid level, AVF CP25 shares 83% identity with the polyprotein of the MsMV1 (query cover 100%, E-value = 2e-126, accession № ATJ00054.1). Noteworthy, less than 90% aa sequence identity among coat protein sequences is another criterion demarcating species in the genus *Marafivirus* [8].

The AVF genome contains a conserved “marafibox”, a 16-nt consensus sequence of the subgenomic RNA (sgRNA) promoter that is present in all known marafiviruses, at positions 5734–5749 nt (5’ GAGGGTGAATTGCTTC 3’). The possible 5’ end of the subgenomic RNA encoding the viral CPs starts at the conserved adenine position 10 nt downstream of the core promoter sequence (A_5759_), [17,18].

Interestingly, the marafivirus-related contigs, assembled from three alfalfa plants collected from the Rhône delta region, shared 75–97% identities with the AVF genome (coverage, 1.e-138< E-value <2.e-9), indicating that these plants were most likely infected with the virus.

### RT-PCR

**Fig 3:**
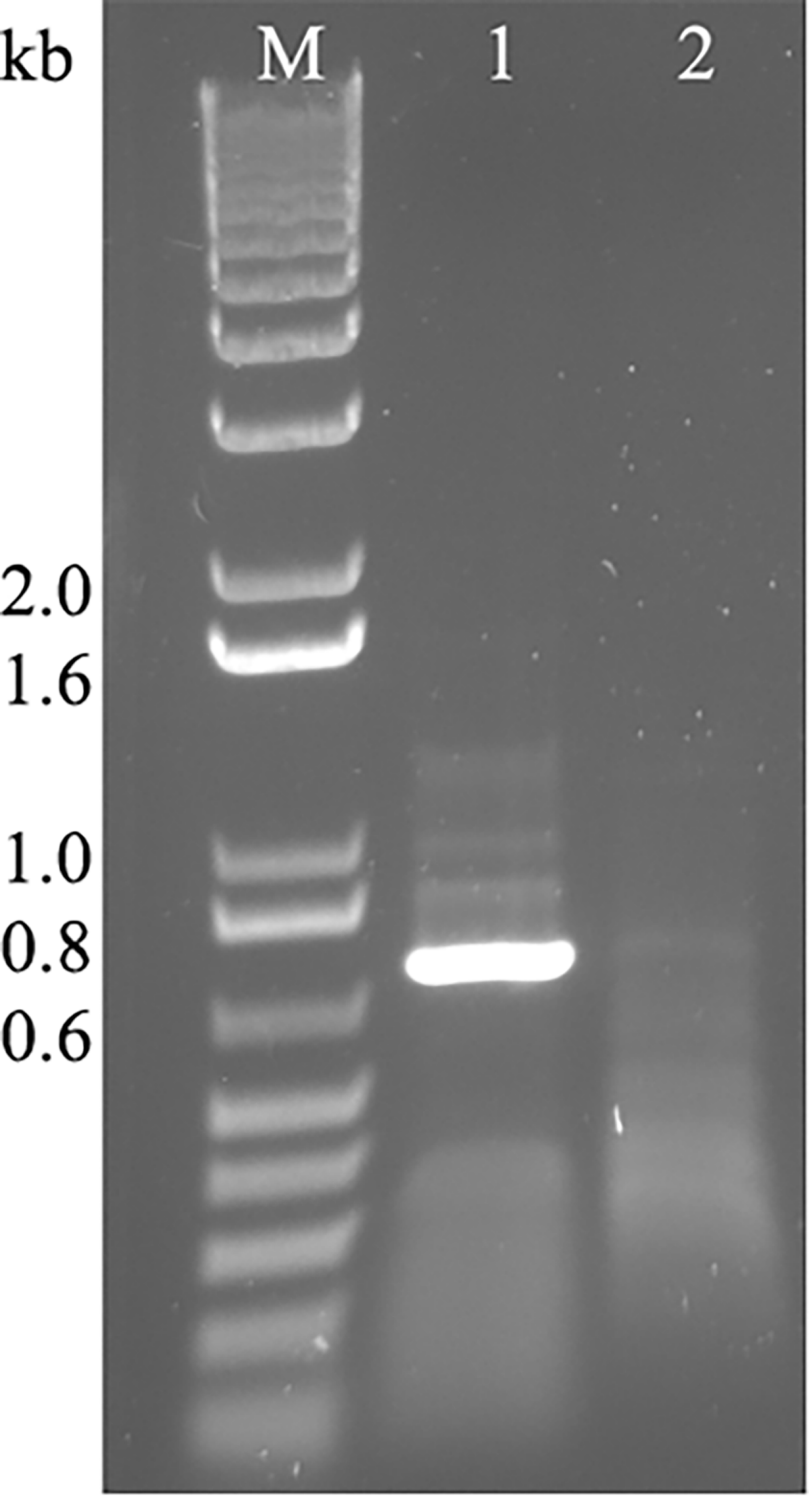
RT-PCR assay with primers LN568-LN569 (Table 1) to amplify a full-length AVF CP25. M, 1kb plus DNA ladder (ThermoFisher Scientific). Lane 1, reaction performed with total RNA extracted from AVF-infected alfalfa sample. Lane 2, RT-PCR with total RNA extracted from healthy alfalfa material.

A complete genome of the AVF obtained via VANA-based Illumina MiSeq and 5’/3’ RACE was used to design primers for RT-PCR amplification of viral sequences encoding the minor CP25 in order to confirm the virus identity and accuracy of the nucleotide sequence. Using primer pair LN568 (forward) and LN569 (complementary) (Table 1) in an RT-PCR assay with total RNA extracted from the infected plant sample, the respective gene was successfully amplified (Fig 3), cloned into the pCRII-TOPO vector (Thermo Fisher Scientific) and sequenced. The nucleotide sequence of the amplified product matched the RNA-seq results thus validating the quality of the digital assembly and the presence of the predicted virus.

### Transmission electron microscopy observations and transient expression of AVF CP

**Fig 4:**
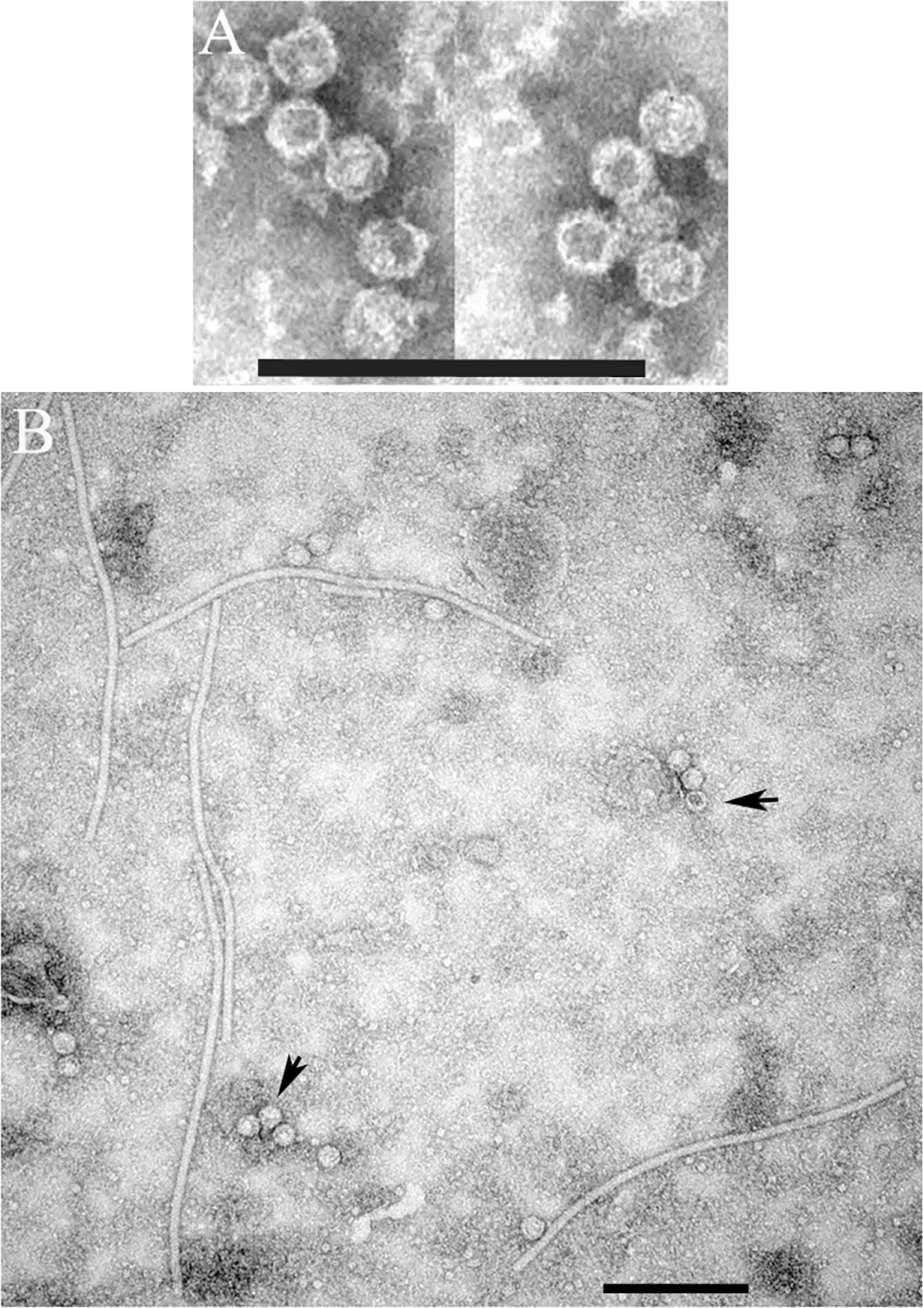
(**A**) Transmission electron microscopy of partially purified alfalfa virus F and (**B**) transient expression of AVF virus-like particles in *Nicotiana benthamiana* plants using a PVX-based vector. Scale bar represents 200 nm. Arrows indicate AVF VLPs.

Routine sample preparation for negative staining [6] did not yield any results. Only when the virus was partially purified as described in [15], spherical virus particles ~ 30 nm in diameter, resembling T = 3 isometric virions of marafiviruses, were observed (Fig 4A).

To confirm functionality of the predicted CP coding region, a fragment of the viral genome encoding the tentative CP25 was transiently expressed in *N*. *benthamiana* plants via a PVX vector [14]. To accomplish this, the RT-PCR product obtained with primers LN568-LN569 designed for amplification of the complete CP25, was sub-cloned into the PVX vector, transcribed and rub-inoculated onto leaves of *N*. *benthamiana*, an experimental host species commonly used in plant virology. Two weeks after inoculation, plants developed symptoms such as stunted growth, vein clearing and chlorotic mosaic (not shown). TEM of crude leaf extracts revealed characteristic PVX rods surrounded by isometric virus-like particles (VLPs) ~ 30 nm in diameter resembling typical marafivirus virions (Fig 4B). Therefore, the proposed sequence of CP25 is accurate and sufficient for assembly of AFV particles. Some virions appeared stain-permeable and others stain-impermeable, suggesting that the latter may encapsidate CP mRNA.

### Phylogenetic analysis

**Fig 5:**
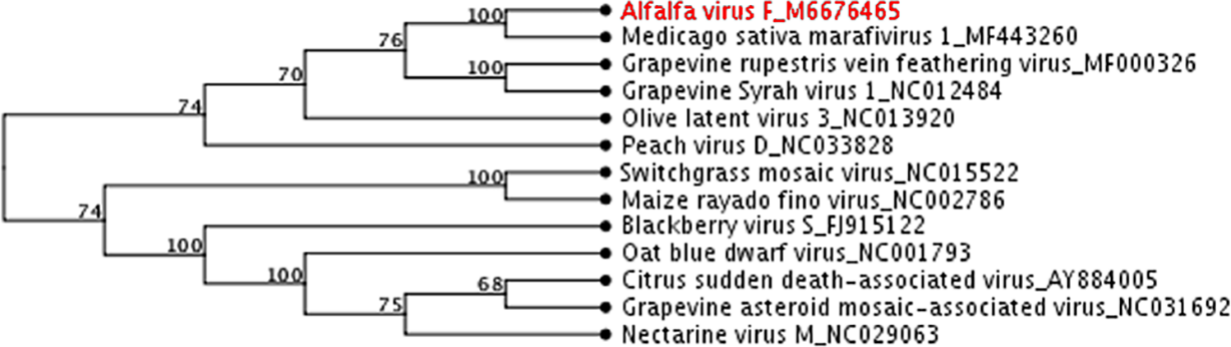
Unrooted neighbor-joining phylogenetic tree built with the complete nucleotide sequences of alfalfa virus F and twelve ICTV-approved and tentative members of the genus *Marafivirus*. The percentage of replicate trees in which the associated taxa clustered together in the bootstrap test (1000 replicates) are shown next to the branches.

A phylogenetic tree was generated with the AVF genome and complete genomic sequences of several ICTV-approved and tentative members of the genus *Marafivirus*.(Fig 5). AVF grouped together with MsMV1 (78% identity vs AVF) and the cluster formed a sister-group with two other ICTV-proposed marafiviruses: grapevine rupestris vein feathering virus and grapevine Syrah virus-1. Both clusters branched out of the larger peach marafivirus D group, connected to the clusters of established species in the genus *Marafivirus*: type member *Maize rayado fino virus*, *Oat blue dwarf virus*, and *Citrus sudden death-associated virus*. Therefore, phylogenetic analysis supported the tentative classification of AFV as a new member of the genus *Marafivirus*.

## Discussion

Here, we reported the discovery of a new alfalfa marafivirus originating from commercial alfalfa fields at Prades-le-Lez, Southern France. Our study indicates that AVF is also present in three alfalfa plants collected in three other areas of Southern France ~75 kms apart from Prades-le-Lez, suggesting that the distribution of AVF in Southern France is probably not restricted to a single area. In this work, a complete genomic sequence of the virus was obtained, identity of the virus was confirmed experimentally and methods suitable for specific diagnostics of the virus were developed. In addition, a portion of the viral genome encoding a minor coat protein (CP25) was transiently expressed in *N*. *benthamiana* plants and successfully assembled into VLPs resembling natural virions, hence validating accuracy of the genome sequence and functional role of the projected CP domain. Since the transiently expressed genome fragment contains information to encode both AVF CP25 and smaller CP21, it is unclear whether the observed VLPs are composed of CP25 alone or of two proteins. If the latter is true, CP25 capsids would have to be post-translationally cleaved with PVX- or host-encoded proteases to form CP21. This, however, requires further experimental confirmation. As previously suggested for MRFV, the ability of transiently expressed AMV CP25 to form VLPs may have potential value for applications in epitope presentation platforms [19].

The first proposed species of the genus *Marafivirus* in alfalfa, MsMV1, was described only recently [11] and before that no members of this genus were identified in alfalfa. Although AVF is most closely related to MsMV1, it does represent a new species, based on the current demarcation criteria in the genus *Marafivirus* [8]. Notably, MsMV1 was not verified in plant samples as it was digitally deduced from alfalfa transcriptomic data published elsewhere and deposited to NCBI GenBank [12]. In addition, judging from the comparison with AVF, the reported sequence of MsMV1 appears to be missing the 5’ terminus and thus its genome length (6675 nt) is most likely inaccurate. Therefore, bioinformatic data alone, without experimental validation, might not be sufficient as the only criteria for characterization of new viral genomes. The final classification of AVF and MsMV1 as new marafivirus species is under consideration by the ICTV *Tymoviridae* study group.

Primary research by Zhang et al. [12], whose transcriptomic data has been later adopted by Kim et. al. [11] for identification of MsMV1, originated from China and employed alfalfa standard varieties Maverick (FDC1) and CUF101 (FDC9) introduced to China from the United States.

Beyond identification and confirmation of AVF as a new viral species infecting alfalfa, the RT-PCR assay with virus-specific primers developed in this work may be of practical use for detection of the virus.

Notwithstanding, we conclude that AVF represents a unique virus species with a close resemblance to the members of the genus *Marafivirus*, family *Tymoviridae* and a more evident relation to the unclassified *Medicago sativa* marafivirus 1. The genome sequence of AVF was deposited in NCBI GenBank on 12/24/2017 as accession № MG676465.

## Supporting information

S1 Fig AMV-infected plant, originating from the Rhône delta region and maintained at the Cirad laboratory. The plant is not exhibiting any visible symptoms that would differentiate it from the healthy plants.
(TIF)
