# Supplementary figures and images for: Characterization of alfalfa virus F, a new member of the genus Marafivirus
Source: PLoS One. 2018 Sep 4;13(9):e0203477. doi: 10.1371/journal.pone.0203477 (PMC6122807; doi:10.1371/journal.pone.0203477)

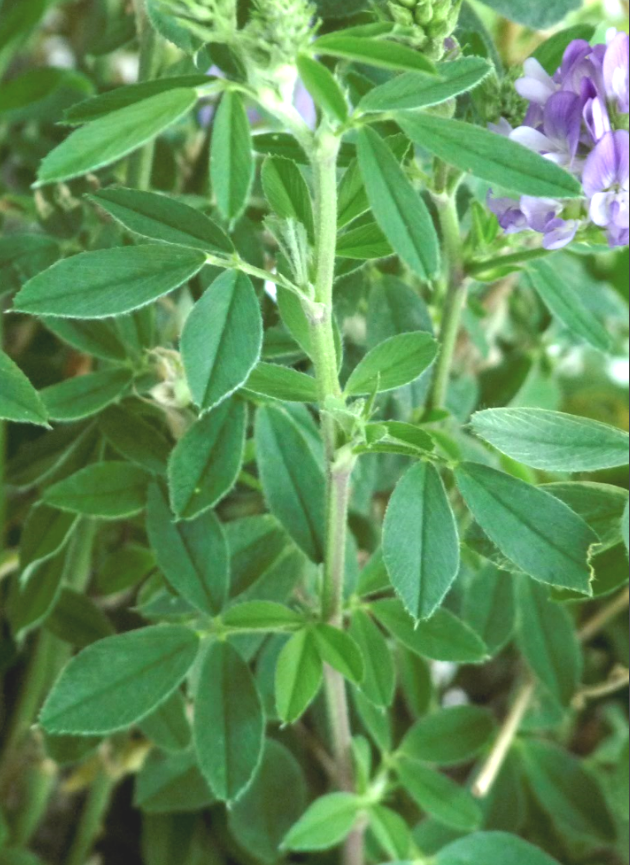

Supplement: S1 Fig — The plant is not exhibiting any visible symptoms that would differentiate it from the healthy plants. (TIF) [file pone.0203477.s001.tif]
